# Learners misperceive the benefits of redundant text in multimedia learning

**DOI:** 10.3389/fpsyg.2014.00710

**Published:** 2014-07-09

**Authors:** Barbara Fenesi, Joseph A. Kim

**Affiliations:** Applied Cognition in Education Lab, Department of Psychology, Neuroscience, and Behavior, McMaster UniversityHamilton, ON, Canada

**Keywords:** instruction, metacognition, learning theory, multimedia, redundant text

## Abstract

Research on metacognition has consistently demonstrated that learners fail to endorse instructional designs that produce benefits to memory, and often prefer designs that actually impair comprehension. Unlike previous studies in which learners were only exposed to a single multimedia design, the current study used a within–subjects approach to examine whether exposure to both redundant text and non-redundant text multimedia presentations improved learners' metacognitive judgments about presentation styles that promote better understanding. A redundant text multimedia presentation containing narration paired with verbatim on–screen text (Redundant) was contrasted with two non-redundant text multimedia presentations: (1) narration paired with images and minimal text (Complementary) or (2) narration paired with minimal text (Sparse). Learners watched presentation pairs of either Redundant + Complementary, or Redundant + Sparse. Results demonstrate that Complementary and Sparse presentations produced highest overall performance on the final comprehension assessment, but the Redundant presentation produced highest perceived understanding and engagement ratings. These findings suggest that learners misperceive the benefits of redundant text, even after direct exposure to a non-redundant, effective presentation.

## Introduction

Lectures and presentations are dominated by the use of multimedia instruction tools such as PowerPoint or Keynote to presumably increase learner attention and engagement (Mantei, 2000; Szabo and Hastings, 2000; Susskind, 2004; Apperson et al., 2008). However, multimedia presentations are often designed ineffectively, leaving audiences disconnected from their learning experiences (Craig and Amernic, 2006; Parker, 2012). Indeed, the pervasive use of redundant text in presentations (i.e., aurally and visually presented verbal information are identical) has been shown to reduce learning (Chandler and Sweller, 1991; Kalyuga et al., 1998, 1999; Fenesi et al., 2014), yet it remains a principle practice. Recent work by Yue et al. (2013) showed that participants prefer identical-full text presentations (paired with images) and think they are best for learning, despite superior learning with presentations containing minimal or no text. However, participants only experienced one condition, and simply indicated which presentation style they would prefer by selecting from a series of answer options [e.g., (a) images and narration only, (b) images, narration, and on-screen text identical to narration]. The current study expanded on prior work by using a within–subjects approach; participants were exposed to redundant and non-redundant text presentations within the same experimental session to determine whether exposure to both presentation styles influenced awareness of the negative effect of redundant text on learning.

There are two dominant instructional theories of multimedia design: Cognitive Load Theory (CLT) (Sweller et al., 1998; Sweller, 1999; Van Merrienboer and Sweller, 2005), and Cognitive Theory of Multimedia Learning (CTML) (Mayer, 2001), both of which propose principles of multimedia design based on the theoretical frameworks of limited working memory capacity (i.e., ability to attend to and process finite information at any given time). Importantly, instructional design that imposes high cognitive load (i.e., when required cognitive processing exceeds working memory capacity) reduces the working memory resources available for processing new information, thus preventing new learning. In addition to its limitations, working memory is comprised of two subsystems for processing information that is auditory/verbal and pictorial/non-verbal (Baddeley, 1986; Paivio, 1986; Chang et al., 2011). Instruction that engages both subsystems to include auditory/verbal information (e.g., narration) and pictorial/non-verbal information can help mitigate limited working memory capacity.

A critical finding shared between CLT and CTML is that information presented through the two subsystems should be complementary rather than identical. If narration is presented with identical written text, the extra verbal information does not provide additional content but merely duplicates the already presented information (Sweller, 1999; Mayer, 2001). The redundant verbal information overwhelms the auditory/verbal subsystem and reduces critical working memory resources needed to meaningfully understand and integrate incoming information. As a result, only simple facts and isolated concepts can be retained and integrated into memory.

However, pairing instructional images with simultaneous auditory narration allows both the pictorial/non-verbal and auditory/verbal subsystems to function in parallel, promoting optimal working memory resource allocation. Also, using images facilitates the construction of mental representations of information, which help integrate new information in working memory with existing long-term memory stores.

Despite clear demonstrations of the negative impact of redundant text on learning (Chandler and Sweller, 1991; Kalyuga et al., 1998, 1999; Adesope and Nesbit, 2012), its use permeates many contexts including education, business and training venues. For instance, among other factors, observers have speculated that poorly designed PowerPoint slides filled with abundant technical information and redundant text may have contributed to complications leading to the 2003 Columbia space shuttle disaster (Tufte, 2003). In addition, an examination of 72 PowerPoint presentations delivered by engineering instructors demonstrated that at least half contained redundant text leading to reduced understanding and retention of key concepts (Gaudelli et al., 2009). These findings are consistent with empirical studies that have compared presentations with redundant text to presentations with narration paired with images, narration paired with minimal text, and narration alone; findings from all studies clearly demonstrate that presentations with redundant text are detrimental to learning (Chandler and Sweller, 1991; Kalyuga et al., 1998, 1999; Fenesi et al., 2014).

Importantly, many studies have incorporated multiple measures of learning, including both comprehension tests and measures of perceived understanding and interest of lecture material (Kalyuga et al., 1998, 1999; Fenesi et al., 2014). Fenesi et al. (2014) recently replicated the negative impact of redundant text on comprehension in conjunction with learners' inability to accurately assess their poor understanding; learners in both the redundant and non–redundant text conditions produced similar perceptions of understanding and interest. These results suggest that learners exposed to redundant text failed to perceive a detriment to objective understanding relative to learners exposed to non–redundant text. Consistent with research in metacognition, these results are not surprising as studies demonstrate learners are typically poor evaluators of their own understanding (Glenberg and Epstein, 1987; Spellman and Bjork, 1992; Benjamin et al., 1998; Kornell and Bjork, 2008).

Metacognitive research investigates learners' abilities to judge their own understanding or skill level (Glenberg and Epstein, 1987; Benjamin and Bjork, 1996). Learners with high metacognitive ability accurately judge their understanding and realize when additional information or practice is required for successful learning. In contrast, learners with low metacognitive ability tend to overestimate their understanding; they often perform poorly on assessments of learning yet perceive that they have successfully mastered the material (Jacoby et al., 1994). Most educational research demonstrates that learners have low metacognitive abilities and typically overestimate their understanding in the absence of actual learning (Glenberg and Epstein, 1987; Spellman and Bjork, 1992; Jacoby et al., 1994; Benjamin and Bjork, 1996; Kornell and Bjork, 2008). Importantly, low metacognitive ability often stems from a sense of familiarity with a particular topic or presentation style; this sense of familiarity is then misattributed to represent comprehension of material in the absence of actual understanding. Findings by Fenesi et al. (2014) that demonstrated a mismatch between perceived understanding and objective comprehension for learners exposed to redundant text presentations may therefore be expected within a metacognitive framework; learners exposed to redundant text were unable to recognize that their understanding was hampered compared to learners exposed to non–redundant text. Additionally, since learners may be more familiar with redundant text presentations due to repeated classroom exposure, they may have inaccurately perceived redundant text to be effective in promoting their understanding.

Previous research assessing the effects of redundant text presentations have typically relied on a between–subjects approach, exposing learners to only one of several possible multimedia presentations. Recent work used this between–subjects approach to demonstrate that participants indicate a preference for redundant text compared to minimal text and images when provided with a list of presentation style options (Yue et al., 2013). However, researchers noted that participants only experienced one condition, which might interfere with accurate metacognitive judgments about the quality of different presentation styles. Therefore, we used a within–subjects approach to examine if learners exposed to both redundant and non-redundant designs within the same experimental session can accurately gauge the differences in learning outcomes elicited between presentation designs.

Learners were presented with two computer–based presentations (one redundant and one non-redundant presentation). The redundant presentation consisted of narration paired with redundant on-screen text. There were two forms of non-redundant presentations; one consisted of narration paired with images and minimal text (Complementary), the other consisted of narration paired with minimal, non–redundant text (Sparse). Therefore, participants were exposed to a combination of either Redundant–Complementary, or Redundant–Sparse presentations, thereby creating experimental conditions: Redundant– Complementary and Redundant–Sparse. The same redundant presentation style was used in both the Redundant–Complementary, and Redundant–Sparse presentations. See Appendix A for visual examples of all three presentation styles. Prior research outlining principles of effective multimedia design demonstrate that Complementary presentations do not overwhelm learners with redundant on–screen text and promote understanding because they contain images that help learners construct mental representations of visual information (Chandler and Sweller, 1991; Kalyuga et al., 1998, 1999, 2000; Tangen et al., 2011; Fenesi et al., 2014). Similarly, Sparse presentations can promote learning because they help focus learner attention on relevant portions of the narration (Mayer and Johnson, 2008; Adesope and Nesbit, 2012; Yue et al., 2013), whereas verbatim redundant text displays all narrative content and overwhelms learners with excessive verbal information. Additionally, learners can engage in unobstructed mental imagery to create mental representations of visual information (Fleming and Hutton, 1983).

Since redundant text reduces learning, we predicted that the Redundant presentation would reduce comprehension performance compared to the Complementary and Sparse presentations. However, we predicted that learners would rate their perceived understanding of material and perceived engagement of the lecture as greater for information learned via the Redundant presentation than via the Complementary and Sparse presentations. These predictions were based on our hypothesis that learners' poor metacognitive judgments (both in terms of perceived understanding and engagement) would be driven by a misattributed sense of familiarity and comfort with redundant text presentations. We also predicted that lecture material interest would be rated equally among presentation styles because previous findings (e.g., Fenesi et al., 2014) found no differences in interest ratings across different presentation styles. Furthermore, we predicted that when learning via the Redundant presentation, learners would rate the perception of lecture difficulty as lower compared to both Complementary and Sparse presentations. This is because learners would equate their sense of familiarity and comfort with Redundant presentations as reduced lecture difficulty.

## Materials and methods

### Participants

Eighty undergraduate students from McMaster University enrolled in the Introductory Psychology course participated in the study in exchange for course credit. Forty participants were randomly assigned to each condition: Redundant–Sparse or Redundant–Complementary. The order of presentation exposure was counterbalanced (i.e., Complementary–Redundant, Sparse–Redundant), with 20 of the 40 participants in each condition assigned to each counter-balanced condition. Participants were drawn from a class of 3000 students consisting of 46% males and 54% females, with a mean age of 19.21 (*SD* = 3.12). Only those without prior (or current) course enrollment in anatomy courses were eligible to participate in the experiment. All participants provided informed consent, and all procedures complied with the tri-council statement on ethics, as assessed by the McMaster Research Ethics Board.

### Materials and procedure

The presentation was displayed on individual 17.5-inch Acer laptops and consisted of a 9-min, system-paced PowerPoint slide show (total of 18 slides) about the physiology, anatomy, evolution, and biochemical mechanisms of hunger. Each presentation style (i.e., Redundant, Complementary, Sparse) was split in half at the 4:30 mark. They were then combined to produce the conditions of interest: Redundant–Complementary, and Redundant–Sparse (counterbalanced: Complementary–Redundant, Sparse–Redundant). The Redundant, Complementary and Sparse presentations had identical audio tracks and only differed in the accompanying visuals. The Redundant presentation consisted of verbatim on–screen text and narration. The Complementary presentation consisted of relevant images (i.e., graphics of the intestinal track were presented during discussion of gastrointestinal chemicals) and minimal, complementary text (i.e., important points succinctly paraphrased). The Sparse presentation was identical to the Complementary presentation but excluded images. At the end of each four–and–a–half minute presentation, learners rated their perceived interest, difficulty, engagement and understanding specific to the preceding presentation. At the end of the experimental session, learners completed a comprehension quiz to assess objective understanding of the information presented. The comprehension quiz tested basic retention of facts (recognition), and deeper conceptual knowledge (applied).

Measures of perceived interest, difficulty engagement and understanding were assessed by a questionnaire following the first and second half of the presentation^1^. Perceived interest was assessed through participants' response to the statement: (1) *I found the material presented in this lecture to be interesting*. Perceived difficulty was assessed through the participants' response to the statement: (2) *The lecture material has a high level of difficulty*. Perceived engagement was assessed through the participants' response to the statement: (3) *I found the multimedia presentation (use of images and/or words) engaging* (engagement). Perceived understanding was assessed through the participants' response to the statement: (4) *I found that I had a meaningful understanding of the material*. All perception responses were made on a 4–point Likert scale (1 = absolutely disagree, 2 = mostly disagree, 3 = mostly agree, 4 = absolutely agree).

Comprehension of the presented material was assessed using a multiple–choice quiz after both of the two presentations were viewed (see Appendix C for comprehension quiz). Principles from Bloom's Taxonomy were used to create distinct recognition and applied questions (Krathwohl, 2010). This allowed us to assess how retention of basic facts (recognition), and the ability to transfer newly learned concepts to novel problem scenarios (applied) were differentially affected by presentation style. A pilot study assessed the reliability of comprehension questions in evaluating recognition vs. applied comprehension. Results showed acceptable internal consistency reliability scores using Kuder–Richardson 20 (Cronbach's alpha reported) for both question types (recognition: K–R 20 = 0.73; applied: K–R 20 = 0.71). In the current experiment 20 comprehension questions (10 recognition, 10 applied) were given, 10 of which tested information from the first half of the presentation, and 10 of which tested information from the second half of the presentation. The number of recognition and applied questions were evenly distributed across both presentations, so that there were five recognition questions and five applied questions for each half of the presentation. The presentations, comprehension questions and perception measures are all available upon request.

An online survey system (Limesurvey) was used to collect responses to the comprehension quiz and the four perception measures. Results were recorded on an anonymous and confidential basis by assigning individual identification numbers. An experimental session lasted 1 h, during which the experimenter was always present.

### Analysis

Comprehension scores were analyzed on SPSS 20 Macintosh using separate 2 (condition: redundant, non-redundant) × 2 (question type: recognition, applied) factorial ANOVAs on each of the non-redundant conditions: Complementary, and Sparse. Paired samples *t-*tests were used to assess differences between specific presentation styles (i.e., Redundant vs. Complementary, Redundant vs. Sparse) on recognition and applied comprehension scores, as well as on ratings of the four perception measures (Norman, 2010), with all pairwise comparisons Bonferroni–corrected to the.05 level. Effect sizes were calculated for main effects, interactions and pairwise comparisons (partial eta squared—η^2^_*p*_—was used for ANOVA, and cohen's *d* was used for paired samples *t-*tests). As results were consistent across counterbalanced conditions, data from the Redundant–Complementary condition were collapsed with data from the counterbalanced Complementary–Redundant condition. This was also true for the Redundant–Sparse and the Sparse–Redundant conditions. The complete data are represented as the Redundant–Complementary, and Redundant–Sparse condition (supplementary material provides for those interested in the counterbalanced conditions and their respective data for comprehension performance and perception measures).

## Results

Comprehension performance and perception measures' ratings are presented in Table 1 for the Redundant–Complementary condition, and in Table 2 for the Redundant–Sparse condition.

**Table 1:** **Mean comprehension performance (recognition, applied) and mean perception measure ratings (interest, difficulty, engagement, understanding) for the Redundant–Complementary condition**.

	**Redundant *M* (*SD*)**	**Complementary *M* (*SD*)**
**COMPREHENSION (%)**		
Recognition	80.5 (7.59)	78.5 (10.89)
Applied	63 (11.28)	76 (9.94)
**PERCEPTION (SCALE 1–4)**		
Interest	3.15 (0.49)	2.9 (0.64)
Difficulty	2.15 (0.59)	3.05 (0.6)
Engagement	3.05 (0.6)	2.25 (0.44)
Understanding	3.15 (0.49)	2.3 (0.57)

**Table 2:** **Mean comprehension performance (recognition, applied) and mean perception ratings (interest, difficulty, engagement, understanding) for the Redundant–Sparse condition**.

	**Redundant *M* (*SD*)**	**Sparse *M* (*SD*)**
**COMPREHENSION (%)**		
Recognition	79 (16.19)	69 (6.41)
Applied	51.5 (15.31)	76 (5.98)
**PERCEPTION (SCALE 1–4)**		
Interest	3.3 (0.66)	2.95 (0.51)
Difficulty	2.15 (0.67)	3.05 (0.39)
Engagement	3.15 (0.93)	2.2 (0.95)
Understanding	3.1 (0.72)	2.35 (0.93)

### Comprehension performance

Analyses for the Redundant–Complementary condition yielded significant main effects of question type, *F*_(1, 19)_ = 16.89, *p* < 0.001, η^2^_*p*_ = 0.47 (performance on recognition questions was greater than performance on applied questions), and condition, *F*_(1, 19)_ = 6.23, *p* = 0.022, η^2^_*p*_ = 0.25 (the Complementary presentation produced greater comprehension than the Redundant presentation). The question type by condition interaction was also significant, *F*_(1, 19)_ = 11.71, *p* = 0.003, η^2^_*p*_ = 0.38. Paired samples *t*-tests yielded no difference in recognition comprehension scores between presentation styles, *t*_(19)_ = 0.68, *p* = n.s., but significantly greater applied comprehension scores in the Complementary presentation compared to the Redundant presentation with a large magnitude–of–effect, *t*_(19)_ = 3.40, *p* < 0.001, *d* = 1.22.

Analyses for the Redundant–Spare condition also yielded significant main effects of question type, *F*_(1, 19)_ = 15.82, *p* < 0.001, η^2^_*p*_ = 0.45 (performance on recognition questions was greater than performance on applied questions) and condition, *F*_(1, 19)_ = 9.63, *p* = 0.006, η^2^_*p*_ = 0.34 (the Sparse presentation produced greater comprehension than the Redundant presentation). The question type by condition interaction was significant, *F*_(1, 19)_ = 35.63, *p* < 0.001, η^2^_*p*_ = 0.65. Paired samples *t*-tests yielded significantly greater recognition comprehension scores for the Redundant presentation compared to the Sparse presentation with a large magnitude–of–effect, *t*_(19)_ = 2.65, *p* < 0.001, *d* = 0.81, whereas applied comprehension scores was significantly greater for the Sparse presentation compared to the Redundant presentation with a large magnitude–of–effect *t*_(19)_ = 6.69, *p* < 0.001, *d* = 0.97.

### Perception measures (interest, difficulty, engagement, understanding)

Perception measures in the Redundant–Complementary condition demonstrate that lecture material was rated as equally interesting between presentations, suggesting that the quality of lecture material was unaffected by differences in multimedia presentation style, *t*_(19)_ = 1.56, *p* = n.s. However, lecture material was rated as significantly less difficult when experienced in the Redundant presentation than in the Complementary presentation with a small to medium magnitude–of–effect, *t*_(19)_ = −5.11, *p* < 0.001, *d* = 0.41. Presentation engagement was rated as significantly greater for the Redundant presentation with a large magnitude–of–effect, *t*_(19)_ = 4.29, *p* < 0.001, *d* = 1.51. Interestingly, perceived understanding ratings were significantly higher for the Redundant presentation compared to the Complementary presentation with a large magnitude–of–effect, *t*_(19)_ = 5.67, *p* < 0.001, *d* = 1.60, despite applied comprehension scores being higher for the Complementary presentation.

Comparisons in the Redundant–Sparse condition were similar to those reported above in the Redundant–Complementary condition. Lecture material was rated as equally interesting between Redundant and Sparse presentations, *t*_(19)_ = 2.10, *p* = n.s. Lecture material was rated as significantly less difficult when experienced in the Redundant presentation than in the Sparse presentation with a large magnitude–of–effect, *t*_(19)_ = 2.11, *p* = 0.003, *d* = 1.64. Presentation engagement was rated as significantly greater for the Redundant presentation with a large magnitude–of–effect, *t*_(19)_ = 3.71, *p* < 0.001, *d* = 1.01. Similar to the Redundant-Complementary condition, perceived understanding ratings were significantly higher for the Redundant presentation compared to the Sparse presentation with a large magnitude–of–effect, *t*_(19)_ = 4.68, *p* < 0.001, *d* = 0.90, despite applied comprehension scores being higher for the Sparse presentation.

## Discussion

The current study examined whether learners recognized the negative impact of a redundant text presentation on objective comprehension when provided with direct comparison to a non-redundant presentation. Results show that although applied comprehension performance was greatest for the non-redundant presentations (i.e., Complementary and Sparse), the Redundant presentation falsely produced greatest perceived understanding. Additionally, the Redundant presentation produced judgments of material difficulty and presentation engagement that did not match objective comprehension performance.

Importantly, this experiment demonstrates that non–redundant presentations produced superior applied comprehension compared to the Redundant presentation. However, the Redundant presentation did not differ in recognition understanding from the Complementary presentation, and produced greater recognition compared to the Sparse presentation. These results however, were not surprising. Redundant presentations may encourage superficial understanding of concepts, but interfere with deeper conceptual understanding due to competing visual–verbal information from on–screen text and narration. Given that recognition understanding evaluates superficial understanding of basic facts and isolated concepts (Jeffries and Maeder, 2006), it is not surprising that Redundant presentations produced similar, if not better, surface knowledge compared to non-redundant presentation styles. On the other hand, learners exposed to effective non-redundant presentations are able to direct greater mental effort to deep, meaningful learning because their attention is not consumed by redundant verbal information. As a result, although non-redundant designs such as the Complementary and Sparse presentations do not enhance recognition performance compared to Redundant presentations, they do promote the transfer of newly learned information to novel problem scenarios, which is vital to effective, long–term learning (Christina and Bjork, 1991).

Interestingly, applied performance of the Redundant presentation style within the Redundant–Sparse condition was significantly less than the applied performance of the Redundant presentation style within the Redundant–Complementary condition. This alludes to the possibility that the Complementary presentation style (but not the Sparse presentation style) influenced the way learners processed and consolidated application-based knowledge that was presented via the Redundant style. Learners may be better able to integrate information across presentation styles when one of the presentations includes helpful images rather than only minimal text. This hypothesis warrants further investigation, and can help contribute to our understanding of why images are highly effective instructional tools.

Redundant text presentations are consistently viewed as positive instructional tools in the absence of meaningful learning. Even when learners were exposed to both redundant and non-redundant presentations within the same session, they were unable to gauge how different presentation styles impacted their objective comprehension. One possibility is that learners may have rated their perceived understanding on a superficial level (i.e., their understanding of basic facts). If this were the case, considering perceived understanding accurately matched recognition comprehension for Redundant presentation styles, it would be highly speculative to conclude that Redundant presentation styles produced poor judgments of understanding. However, learners were encouraged to judge their understanding beyond basic fact recognition by rating whether they had a meaningful understanding of the lecture. Another potential limitation of the current study is the exclusion of a presentation condition with both redundant text *and* images. However, extensive prior research comparing presentation styles containing images without redundant text (i.e., Complementary) to presentation styles containing images with redundant text, have demonstrated that the presence of redundant text significantly reduced comprehension (Kalyuga et al., 1999; Mayer et al., 2001; Mayer and Moreno, 2002). Overall, our results demonstrate that although Redundant presentations reduce meaningful learning, they are perceived as overwhelmingly positive. This supports our predictions that even when learners are exposed to both redundant and non-redundant presentations, they perceive redundant text as a superior instructional tool.

Our results also relate to the concept of *desirable difficulties*—where difficulty during initial learning yields better long–term retention than initial learning that is effortless (Bjork, 1994). Prior research on desirable difficulties has demonstrated that learners who are required to manipulate information in an initially complex, meaningful way (i.e., generating mnemonics, learning words inverted) compared to learners who simply memorize information, perform poorly on immediate comprehension tests; however, the same learners show superior retention on delayed comprehension tests (Bjork, 1994; Sungkhasettee et al., 2011). Our results also coincide with the concept of desirable difficulties, as perceptions of greater difficulty (as seen in the Complementary and Sparse presentations) corresponded with superior comprehension performance compared to lower ratings of difficulty (as seen in the Redundant presentation).

Our findings may also reflect the concept of *amount of invested mental effort* (AIME) (Salomon, 1983). AIME suggests that learners invest different amounts of mental effort depending on the situation. In situations when learning is perceived as fluid and easy (akin to Redundant presentations), less mental effort is expended to engage with the content. In contrast, when learning is perceived as more difficult, more mental effort is invested to understand content. Crucially, AIME posits that the amount of mental effort expended during learning has a direct impact on how well something is learned. As a result, since the Redundant presentation was perceived as less difficult, participants potentially exerted less mental effort during acquisition of presented information, consequently reducing meaningful learning. Since only minimal mental effort is required to understand basic facts (i.e., recognition knowledge), the Redundant presentation still encouraged recognition-based learning, but failed to evoke the necessary mental effort needed to produce high-level, application-based knowledge.

## Conclusions

The results of the current study extend the metacognitive literature by demonstrating that learners are poor judges of their own understanding in an educational context. Repeated exposure to Redundant presentations in an educational context may instill a sense of familiarity that learners misinterpret as representing effective multimedia instruction. Importantly, inaccurate metacognitive judgments are so robust that exposure to effective and ineffective multimedia instruction could not produce appropriate assessments of perceived interest and understanding that were in–line with objective comprehension performance. As a result, Redundant presentations may pervade educational institutions because learners reinforce instructors to use redundant text (based on misguided perceptions of familiarity) and instructors correspondingly seek to appease student demands. Moreover, instructor evaluations, which rely heavily on student satisfaction (Marsh, 1980; Greenwald, 1997), may reinforce the use of redundant text in presentations to ensure students' sense of familiarity and comfort resulting in a debilitating cycle of ineffective instructional design. This cycle may lead learners to reject the use of more effective presentation designs with limited use of redundant text. It is also possible that students are accustomed to factual learning, which is often aided by rote-memorization of verbatim notes. Redundant presentations clearly promote factual knowledge (i.e., recognition knowledge), and are therefore well-matched to rote-memorization of fact-based information. It would be interesting if future work investigated whether there is a relationship between preference for Redundant presentations and the experience of classroom techniques employing rote-memorization.

There are several interventions that can be employed to avoid the learning pitfalls of redundant text presentations. First, instructors need access to appropriate multimedia training to avoid reliance on redundant text and promote the use of relevant images and minimal text. Making instructors aware of the cognitive detriments of redundant text may augment such practical training. Perhaps the overuse of redundant text in instructional design is in large part due to a lack of instructor awareness regarding effective and ineffective use of text and images during instruction. By educating instructors on appropriate multimedia design, they can in turn communicate to students why redundant text, although subjectively preferred, is an ineffective learning design. This flow of information is critical to diffusing the detrimental student–instructor cycle characterized by a sequence of student preference for redundant text, and instructor appeasement for student satisfaction. Second, as learners become regularly exposed to effective presentations in educational settings, they may develop an appropriate sense of familiarity with presentations that facilitate their understanding. It is critical that research continues to investigate ways to align objective, successful learning with subjective perceptions of understanding to maximize student achievement.

### Conflict of interest statement

The authors declare that the research was conducted in the absence of any commercial or financial relationships that could be construed as a potential conflict of interest.

## Footnotes

^1^Although some researchers encourage using multiple items to measure a single construct (e.g., perceived difficulty), there is extensive research demonstrating that single items (e.g., using one perception item to measure perceived difficulty) can adequately measure a given construct (Gardner et al., 1998; Bergkvist and Rossiter, 2007). As a result, we adopted the approach of using single perception items to measure the subjective constructs of interest, difficulty, engagement and understanding.
