# Supplementary material for: Learners misperceive the benefits of redundant text in multimedia learning
Source: Front Psychol. 2014 Jul 9;5:710. doi: 10.3389/fpsyg.2014.00710 (PMC4088922; doi:10.3389/fpsyg.2014.00710)
Supplement: Supplementary file 1 [file Presentation1.PDF]

Appendix A

Examples of Presentation Styles

Redundant Condition

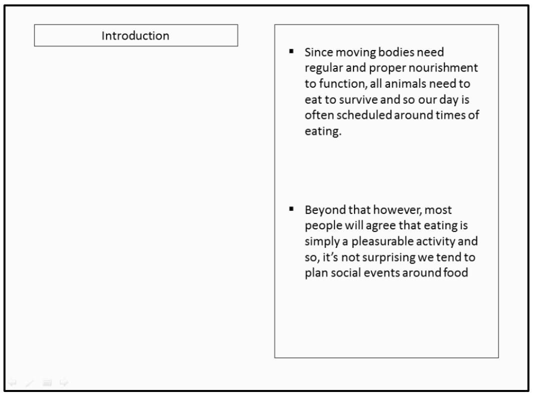

Complementary Condition

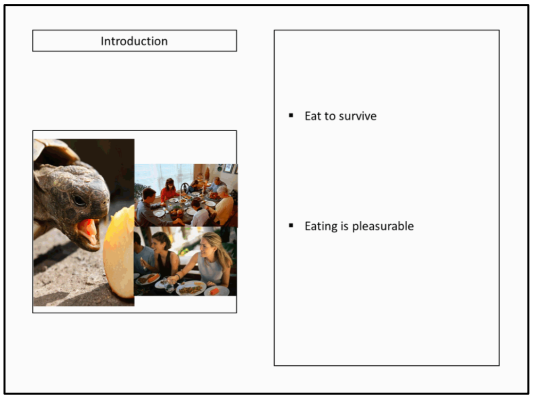

Sparse Condition

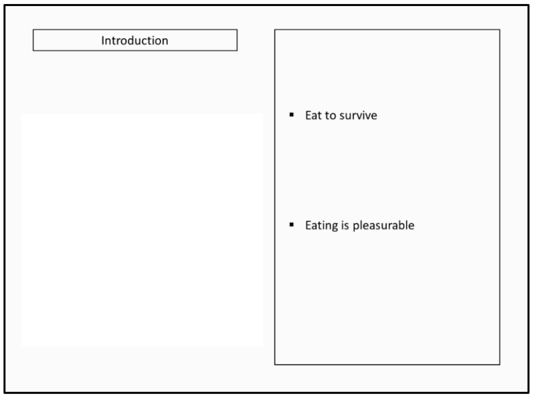

Appendix B

Counterbalanced data for comprehension scores and perception measures

Table 3. Comprehension scores for all counterbalanced presentations.

Comprehension Question (%)	Presentation style *M (SD)*	
	**Redundant**	**Complementary**
**Recognition**	80 (10.54)	76 (12.65)
**Applied**	66 (13.50	77 (11.60)
	**Complementary**	**Redundant**
**Recognition**	81 (8.76)	81 (3.16)
**Applied**	75 (8.50)	60 (8.16)
	**Redundant**	**Sparse**
**Recognition**	79 (15.24)	70 (6.67)
**Applied**	50 (6.67)	75 (5.27)
	**Sparse**	**Redundant**
**Recognition**	68 (6.32)	79 (17.92)
**Applied**	77 (6.75)	53 (21.11)

Table 4. Perception measures for all counterbalanced presentations.

Perception (scale 1-4)	Presentation style *M (SD)*	
	**Redundant**	**Complementary**
**Interest**	3.2 (0.42)	2.9 (0.57)
**Difficulty**	1.8 (0.42)	3 (0.47)
**Engagement**	3.1 (0.57)	2.3 (0.48)
**Understanding**	3.2 (0.42)	2.3 (0.68)
	**Complementary**	**Redundant**
**Interest**	2.9 (0.74)	3.1 (0.57)
**Difficulty**	3.1 (0.74)	2.5 (0.53)
**Engagement**	2.2 (0.42)	3 (0.67)
**Understanding**	2.3 (0.48)	3 (0.57)
	**Redundant**	**Sparse**
**Interest**	3.4 (0.52)	3.2 (0.42)
**Difficulty**	1.7 (0.48)	2.9 (0.32)
**Engagement**	3.1 (1.20)	2.2 (1.23)
**Understanding**	3.2 (0.79)	2.7 (1.06)
	**Sparse**	**Redundant**
**Interest**	2.7 (0.48)	3.2 (0.79)
**Difficulty**	3.2 (0.42)	2.6 (0.52)
**Engagement**	2.2 (0.63)	3.2 (0.63)
**Understanding**	2 (0.67)	3 (0.67)

Appendix C

Comprehension Questions

First half of presentation: Recognition questions (correct answers indicated in bold)

Cannon and Washburn (1992) proposed an interesting answer to why people feel hungry. What answer did they purpose?

a. You feel hungry when the walls of your intestine rub against each other
b. You feel hungry when the glucose in your stomach causes a sensation of emptiness
c. You feel hungry when the walls of your stomach rub against each other
d. You feel hungry when your small intestine contracts, and sends digestive enzymes up to your stomach

In 1944, Inglefinger studied cancer patients with their stomachs surgically removed. Describe what his study concluded about feelings of hunger?

a. You need a stomach in order to feel hungry
b. You do not need a stomach in order to feel hungry
c. Individuals without stomachs do not report feelings of hunger
d. You need only part of your stomach to feel hungry

As glucose levels drop:

a. You start feeling full
b. Remaining glucose is quickly converted into glycogen
c. Glycogen is broken down into glucose
d. Fat is stored

Which nutrient signals the need to replenish one's food intake?

a. glucose
b. fructose
c. adipose tissue
d. glycogen

According to the lecture, "our lives seem dominated by the consumption of food". What was the evolutionary rationale behind this statement?

a. In the past, humans had to expend more effort in order to find food than is typical for modern industrial societies today.
b. In the past, humans had to expend minimal effort in order to find than is typical for modern industrial societies today.
c. In the past and in present industrial societies, humans expend a great deal of energy seeking out scarcely available food.
d. In the past and in present industrial societies, humans expend less energy seeking out scarcely available food.

First half of presentation: Applied questions

You have discovered an animal that does not seem to employ glycogen stores (or an equivalent). Applying your knowledge about glycogen stores, you might expect this animal to:

a. **Eat frequently and have highly variable glucose levels**
b. Eat frequently and have consistently low glucose levels
c. Eat infrequently and have highly variable glucose levels
d. Eat infrequently and have consistently low glucose levels

Peter's liver is correctly identifying glucose levels in his blood. Frank's liver is incorrectly identifying glucose levels in his blood. What would be the difference between Peter and Frank's liver activity?

a. **Peter's but not Frank's liver would be breaking down glycogen into glucose when glucose levels are low**
b. Frank's but not Peter's liver would be breaking down glycogen into glucose when glucose levels are low
c. Peter's but not Frank's liver would be converting glucose into glycogen when glycogen levels are low
d. Frank's but not Peter's liver would be converting glucose into adipose tissue when glycogen levels are low

In a transporter malfunction, John's stomach was accidently removed. What effect will it have on his eating habits?

a. John will eat more
b. John will eat slightly less
c. John will now experience highly variable levels of hunger
d. **No effect**

Jim has a rare autoimmune disorder where his fat tissues attack his muscle cells. What kind of side effects would Jim experience as a result of his disorder?

a. **High blood glucose**
b. Low blood glucose
c. Fatty acids start breaking down glycogen
d. Fatty acids start breaking down glucose

Alex, Sam, and Amanda all eat the same amount of food for dinner. Alex eats a triple cheeseburger with fries. Sam eats a large turkey sandwich with a plate of steamed vegetables. Amanda eats a large plate of chicken thighs and wings. Who will feel full the longest?

a. Alex
b. Amanda
c. **Sam**
d. They will all feel full for the same amount of time

Second half of presentation: Recognition questions

Physiological evidence indicates that part of the _____ controls the cessation of feeding. It appears to do so by ______.

a. hypothalamus…monitoring stomach distension
b. thalamus…monitoring the rate of glucose use
c. hypothalamus…monitoring the rate of glucose use
d. limbic system…monitoring the rate of glucose use

According to the lecture, which part of the brain is the most important in the regulation of hunger and satiety?

a. olfactory bulb
b. prefrontal cortex
c. hippocampus
d. hypothalamus

Damage to the brain area important in regulating eating behaviour can affect hunger and satiety in two different ways. What are they?

a. overeating only
b. refusing to eat only
c. overeating, or refusing to eat
d. emotional overeating only

Stimulation of the ventromedial nucleus of the hypothalamus in rats might be expected to cause:

a. an increase in food intake and weight gain
b. a sharp decrease in food intake (or its complete cessation) and weight loss
c. a transition from waking to sleep, if the stimulation is of high frequency
d. permanent wakefulness

What is the role of adipose tissue?

a. stores energy for later use
b. signals the body to replenish its food intake
c. maintains the body at a healthy weight
d. carries glucose to different areas of the body

Second half of presentation: Applied questions

Dr. Smith discovers one of his patients (Mike) has been gaining weight. Upon closer inspection, Dr. Smith discovers Mike's leptin levels are abnormally low. What role does leptin play in long-term weight regulation?

a. When fat tissue increases, leptin production is halted, and daily food consumption is lowered
b. When an individual feels hungry, leptin levels rise, and signal the body to consume food
c. When an individual feels hungry, leptin levels rise, and signal the body to reduce food consumption
d. When fat tissue increases, leptin levels rise, and is involved in reducing daily food consumption.

A pharmaceutical company is trying to create a drug that will help obese clients lose weight. Taking advantage of what you have learned thus far, which of the following approaches would be best?

a. Create a drug that mimics the function of Leptin
b. Create a drug that stimulates the liver to break down glycogen to glucose
c. Create a drug that blocks the receptors of NPY
d. Create a drug that stimulates the overproduction of adipose tissues

Dr. Smith discovers the presence of a hormone in the small intestine, which he hypothesizes, causes feelings of fullness, and reduces eating. Which following observation (if found) would argue against his hypothesis?

a. When this hormone was injected into subjects, it resulted in feelings of nausea. Therefore, perhaps nausea, and not a feeling of fullness, reduced food consumption
b. When this hormone was injected into subjects, it caused stomach constriction and intense gastrointestinal pain. Therefore, perhaps pain, and not a feeling of fullness, reduced food consumption.
c. When this hormone was injected into subjects, it caused stomach the esophagus to constrict, consequently preventing food consumption. Therefore, perhaps esophagus constriction, and not a feeling of fullness, reduced food consumption.
d. When this hormone was injected into subjects, feelings of fatigue resulted. Therefore, perhaps fatigue, and not feelings of fullness, reduced food consumption.

John acquires a head injury during a car accident and over the subsequent weeks he gains over 80 pounds. What may have been the cause for his excessive weight gain?

a. Overproduction of CCK in the brain
b. Lateral hypothalamus damage
c. Ventromedial hypothalamus damage
d. Damage to hypothalamus inhibiting production and release of NPY

Dr. Burn has discovered a new hormone called DBH that he believes directly inhibits the actions of NPY. Which of the following experimental procedures would allow Dr. Burn to test his hypothesis?

a. Inject DBH into the <u>hypothalamus</u>; if eating <u>increases,</u> his hypothesis is correct

b. **Inject DBH into the hypothalamus; if eating decreases, his hypothesis is correct**
c. Inject DBH into the liver; if eating increases, his hypothesis is correct
d. Inject DBH into the liver, if eating decreases, his hypothesis is correct
